# Navigation-assisted suture anchor insertion for arthroscopic rotator cuff repair

**DOI:** 10.1186/s12891-019-3021-2

**Published:** 2019-12-29

**Authors:** Ivan Micic, Erica Kholinne, Hanpyo Hong, Hyunseok Choi, Jae-Man Kwak, Yucheng Sun, Jaesung Hong, Kyoung-Hwan Koh, In-Ho Jeon

**Affiliations:** 1 Clinic for Orthopaedic Surgery and Traumatology, Clinical Center Nis, Nis, Serbia; 2 Department of Orthopedic Surgery, Asan Medical Center, College of Medicine, University of Ulsan, Seoul, South Korea; 3 Department of Orthopedic Surgery, St. Carolus Hospital, Jakarta, Indonesia; 4 Department of Robotics Engineering, Daegu Gyeongbuk Institute of Science and Technology, Daegu, South Korea; 5 Department of Hand Surgery, Affiliated Hospital of Nantong University, Nantong, Nantong University, Nantong, Jiangsu China

**Keywords:** Navigation, Arthroscopic, Subscapularis, Anchor, Location, Angle

## Abstract

**Background:**

Suture anchor placement for subscapularis repair is challenging. Determining the exact location and optimum angle relative to the subscapularis tendon direction is difficult because of the mismatch between a distorted arthroscopic view and the actual anatomy of the footprint. This study aimed to compare the reliability and reproducibility of the navigation-assisted anchoring technique with conventional arthroscopic anchor fixation.

**Methods:**

Arthroscopic shoulder models were tested by five surgeons. The conventional and navigation-assisted methods of suture anchoring in the subscapularis footprint on the humeral head were tested by each surgeon seven times. Angular results and anchor locations were measured and compared using the Wilcoxon signed rank test. Interobserver intraclass correlation coefficients (ICCs) were analyzed among the surgeons.

**Results:**

The mean angular errors of the targeted anchor fixation guide without and with navigation were 17° and 2° (*p* < 0.05), respectively, and the translational errors were 15 and 3 mm (p < 0.05), respectively. All participants showed a narrow range of anchor fixation angular and translational errors from the original target. Among the surgeons, the interobserver reliabilities of angular errors for ICCs of the navigation-assisted and conventional methods were 0.897 and 0.586, respectively, and the interobserver ICC reliabilities for translational error were 0.938 and 0.619, respectively.

**Conclusions:**

The navigation system may help surgeons be more aware of the surrounding anatomy and location, providing better guidance for anchor orientation, including footprint location and anchor angle.

## Background

The subscapularis muscle is the largest and most powerful rotator cuff muscle, which is important in shoulder joint movement and mechanics [1]. The characteristics of tears in the subscapularis make arthroscopic repair challenging, particularly due to the presence of adjacent neurovascular structures and limited working area [2]. Furthermore, intraoperative assessment of the exact placement of the suture anchor for the subscapularis tendon repair is difficult [1, 2]. This is likely caused by the mismatches between the exact orientation of the subscapularis fibers and distortion of the arthroscopic image. Although insertion of a 70**°** arthroscope through the posterior portal can improve the visualization of the subscapularis tendon footprint, it can also mislead the subscapularis anatomy. Thus, only a limited part of the footprint can actually be exposed.

Moreover, recognizing the orientation of suture anchor insertion is difficult. First, to secure satisfactory anchor placement, Burkhart et al. recommended that the anchors be advanced via the anterior portal, with the surgeon’s hand at the patient’s chin for proper anchor orientation [3]. Second, the position of the 70° arthroscope and distortion resulting from its camera lens make the surgical procedure more challenging. Third, arthroscopic determination of the subscapularis tendon fibers is more difficult than determination of the supraspinatus tendon fibers because the former run differently from the lesser tuberosity, whereas the latter can easily be identified on the greater tuberosity.

Suture anchor insertion at the optimum angle is essential to secure the rotator cuff at the footprint [4], resulting in consistent repair of the subscapularis. Various studies have suggested that the dead-man angle is the optimum angle for the suture anchor insertion procedure [5–8]. However, the reference for this optimum angle remains debatable. The dead-man angle is believed to represent the Newtonian physics of strength required to provide an adequate force to hold the tissue in the footprint until the tendon heals [5–7]. Nevertheless, in the absence of a clear reference, the placement and maintenance of the suture anchor in accordance with the dead-man angle show inadequate reliability and reproducibility. Moreover, the validity of the dead-man angle theory in a real rotator cuff repair situation has been questioned [4, 8, 9].

Regardless of the uncertainty of anchor insertion angle, this study focused solely on measuring the actual anchor angle and location, and its accurate guidance. This study aimed to (1) assess the accuracy and reliability of the designated angle and location of suture anchor insertion into the humeral head for subscapularis repair, comparing the navigation and conventional techniques in the experimental laboratory setting and (2) assess whether the navigation-assisted surgical system allowed the surgeons to customize their preferred target angle and location. We hypothesized that use of the surgical navigation system would result in more accurate and reliable suture anchor placement than the use of the conventional method.

## Methods

The study used arthroscopic models of the shoulder joint (Arthrex, USA) which has been used in several studies [10–12]. The models were scanned by three-dimensional computed tomography, and reconstructed for navigation. The navigation system to tracker was subsequently calibrated. The reference marker of the navigation tracker was physically attached to the bone of the shoulder models, and the five fiducial markers were also built in the models for the patient-to-image registration. The fiducial registration error was 0.55 mm. After completing the patient-to-image registration using by the markers, we can access any anatomical points. Including the lesser tuberosity as our target point.

**Fig. 1:**
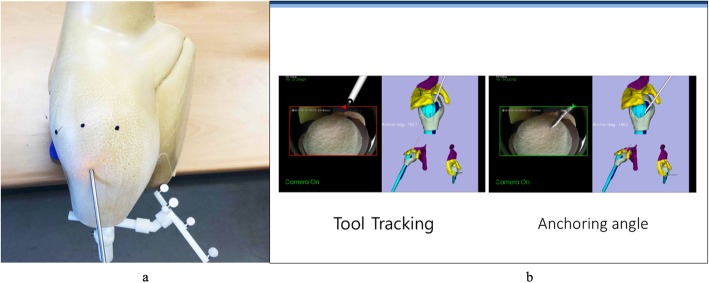
The plastic model (Arthrex, USA) with humerus marker (**a**). Intraarticular view from navigation experiment to show real-time information regarding tool tracking and anchoring angle (**b**)

**Fig. 2:**
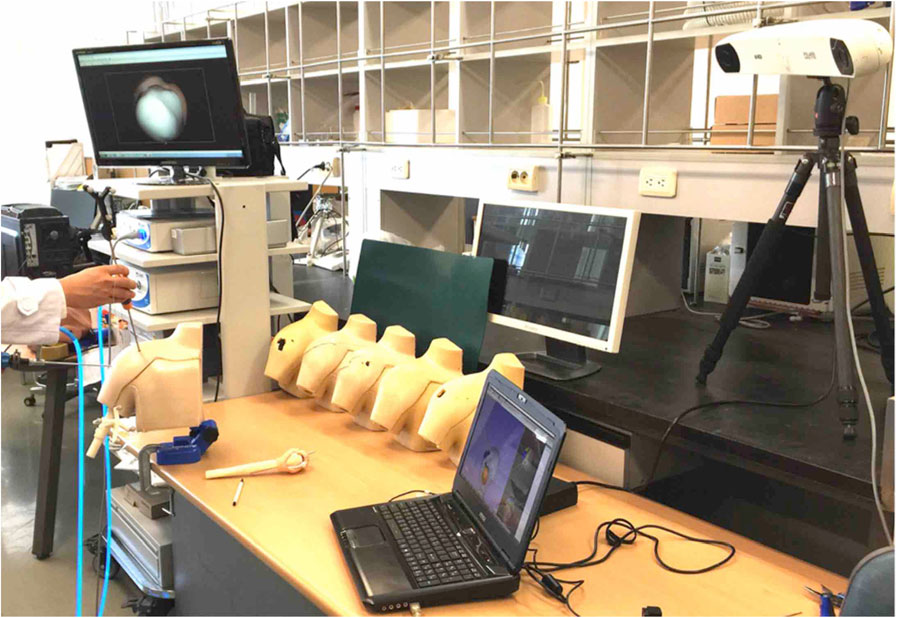
Laboratory setting of navigation and arthroscopy

**Fig. 3:**
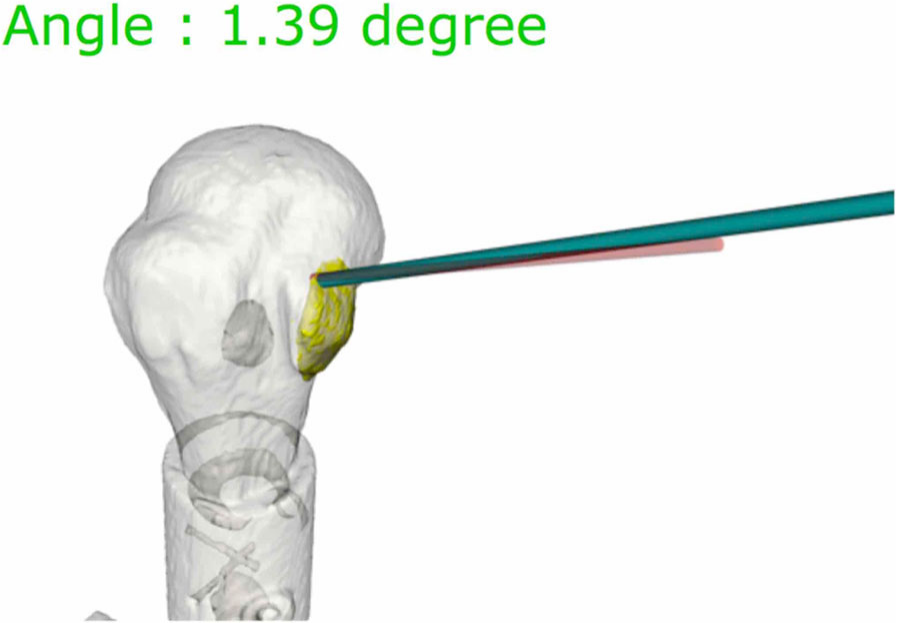
Preoperative anchor target (red rod) with real-time anchor position and angular error (green rod) from the target

The arthroscopic shoulder model were tested by five surgeons. The participating surgeons were novices with limited experience on shoulder arthroscopic surgery. Before surgery, all participants were shown the designated anchor position and orientation on three-dimensional models of the shoulder (Arthrex, USA). The posterior portal was used to view subscapularis repair, thus establishing a standard gleno-humeral view. The footprint on the lesser tuberosity was determined. The tap for the pilot hole of the suture anchor (Helicoil® Regenesorb 5.5-mm suture anchor, Smith & Nephew, MA, USA) was inserted through the anterior portal (Figs. 1, 2). All participants performed the experiment seven times, each using the conventional patient chin-oriented method of placing the anchor in the subscapularis footprints on the humeral head. [1, 2] Before the experiment, the target position **p**_*t*_ and orientation R_*t*_ of the anchor were determined by a senior surgeon and used as a reference for comparison (Fig. 3). When anchoring was finished, position **p**_*i*_ and orientation R_*i*_ of the anchor were measured using the navigation tracker (Polaris spectra, NDI Inc., Canada). The orientation symbol R was represented as a 3 × 3 matrix, which was built from the XYZ Euler angle. Position **p** was a three-component vector composed of the x, y, and z coordinates of the anchor. The difference between the measurement [R_*i*_| **p**_*i*_] and target poses [R_*t*_| **p**_*t*_] of the anchor was calculated. The angle *θ* between the target and measured poses could be calculated as follows:

$$ {\mathbf{v}}_t={\mathrm{R}}_t\bullet {\left[0\kern0.5em 0\kern0.5em 1\right]}^T $$

$$ {\mathbf{v}}_i={\mathrm{R}}_i\bullet {\left[0\kern0.5em 0\kern0.5em 1\right]}^T $$

$$ \theta =\frac{{\left\Vert {\mathbf{v}}_t\times {\mathbf{v}}_i\right\Vert}_2}{\left|{\mathbf{v}}_t\bullet {\mathbf{v}}_i\right|} $$

The difference *d* between the two positions was calculated as a second norm, which represents a scalar distance.

$$ d={\left\Vert {\mathbf{p}}_i-{\mathbf{p}}_t\right\Vert}_2 $$

The same procedures were performed seven times, with the assistance of navigation for anchor insertion. Each time fixation was finished, the angle (angular error) *θ* and distance (translational error) *d* were measured.

At the end of the experiment, the surgeons rated the utility of the navigation-assisted arthroscope system in aiding suture anchor insertion using a Likert scale questionnaire system (very useful, useful, or not useful).

### Statistical analyses

The median values of the angular and locational differences of insertion from the designated angle and location were calculated and compared using Wilcoxon signed rank tests. The significance level and intra- and interobserver reliabilities analyzed using repeated measure analysis of variance were used to calculate intraclass correlation coefficients (ICCs) and their 95% confidence intervals [13–15]. ICC values of > 0.81, 0.61–0.80, 0.41–0.60, 0.21–0.40, and 0.00–0.20 were considered almost perfect, substantial, moderate, fair, and poor, respectively [16].

## Results

### Angular and translational errors

**Fig. 4:**
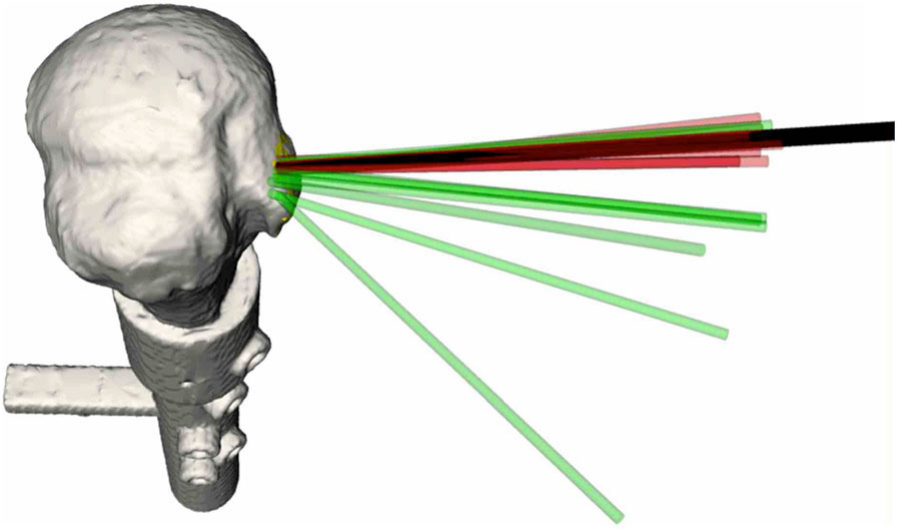
Preoperative anchor target (black), with [17] and without (green) navigation assistance

**Fig. 5:**
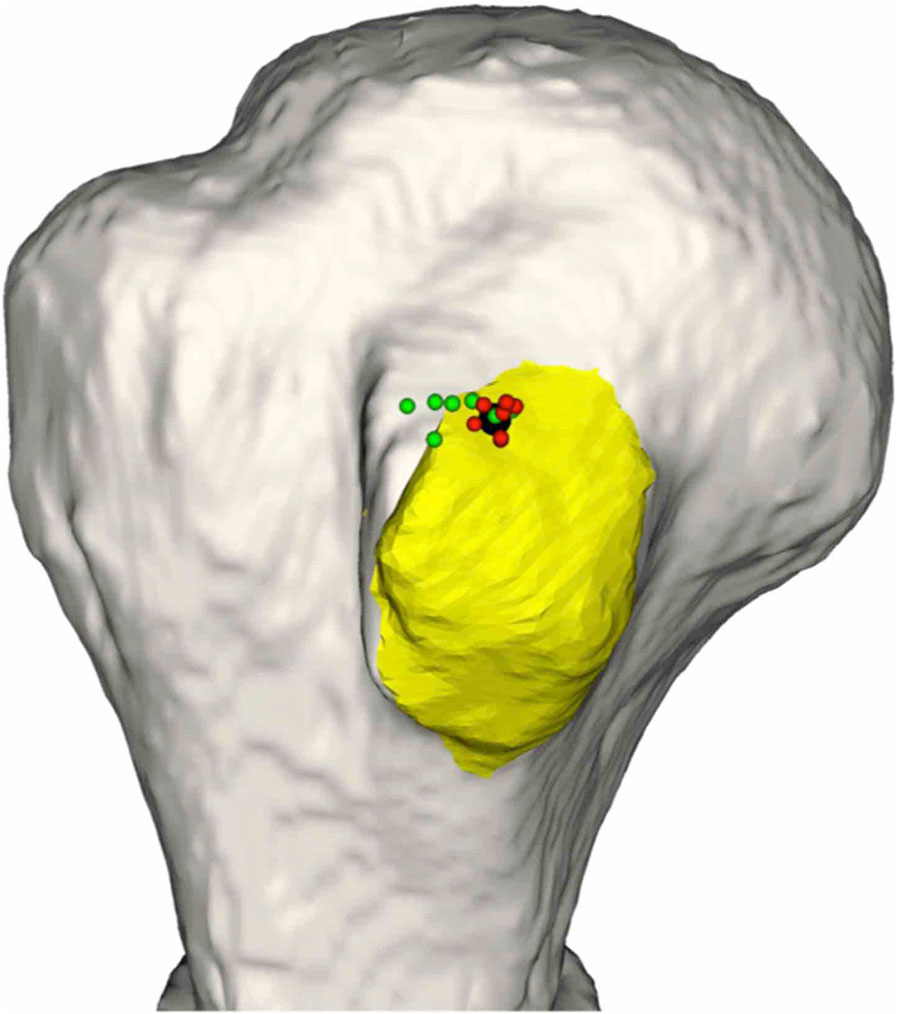
Target point (black), with [17] and without (green) navigation assistance

The mean angular errors from the targeted anchor fixation guide without and with navigation were 17° and 2° (*p* < 0.05), respectively (Fig. 4), indicating the usefulness of the navigation system in determining angular guidance that can be set by the surgeons. The translational errors without and with navigation were 15 and 3 mm (p < 0.05), respectively (Fig. 5). Two different methods could be visually clarified by recording the anchor location. Translational errors were significantly reduced by the navigation system, and, similarly in the pistol group analysis, it is advantageous in terms of accuracy. All participants rated the navigation system as very useful. In addition, the participants showed narrower ranges of anchor fixation angular error and anchor translational error relative to the original target (Table 1).

**Table 1:** Comparison between conventional and NAS methods in terms of accuracy

Investigator #	Angular error (degree)		Translational error (mm)	
	Conventional	NAS	Conventional	NAS
1	12	1	4	2
2	28	2	20	3
3	14	1	17	2
4	15	2	15	3
5	15	1	21	3
Average	17	2	15	3
*SD*	9.8	1.3	9.04	2
*P-*value (independent Student’s t-test)	0.0001		0.0001	
*P-*value (paired Student’s t-test)	0.0001			

### Interobserver and intraobserver reliability

The interobserver reliability of ICCs showed moderate-to-almost perfect (0.477–1.000) and slight-to-almost perfect (0.010–0.898) values using the navigation system and conventional method, respectively (Table 2). ICC of the angular error was lower than that of the translational error, particularly using the conventional method (Table 2). The intraobserver reliability of ICCs using the navigation-assisted and conventional error methods were 0.897 and 0.586 for angular error and 0.938 and 0.619 for translational, respectively (Table 3). ICCs showed that the navigation-assisted method was more reliable for anchor fixation than the conventional method.

**Table 2:** Interobserver reliability ICC of both methods in terms of angular and translational errors

*Conventional*								
angular error	Investigator 2		Investigator 3		Investigator 4		Investigator 5	
Investigator 1	0.405	fair	0.715	substantial	0.045	slight agreement	0.010	slight agreement
Investigator 2			0.732	substantial	0.725	substantial	0.615	substantial
Investigator 3					0.577	moderate	0.167	slight agreement
Investigator 4							0.436	moderate
translational error	Investigator 2		Investigator 3		Investigator 4		Investigator 5	
Investigator 1	0.756	substantial	0.761	substantial	0.898	almost perfect	−0.396	slight agreement
Investigator 2			0.722	substantial	0.800	substantial	−0.873	slight agreement
Investigator 3					0.878	almost perfect	−0.580	slight agreement
Investigator 4							−0.607	slight agreement
*NAS*								
angular error	Investigator 2		Investigator 3		Investigator 4		Investigator 5	
Investigator 1	0.883	almost perfect	1.000	almost perfect	0.742	substantial	0.879	almost perfect
Investigator 2			0.883	almost perfect	0.864	almost perfect	0.979	almost perfect
Investigator 3					0.742	substantial	0.890	almost perfect
Investigator 4							0.773	substantial
translational error	Investigator 2		Investigator 3		Investigator 4		Investigator 5	
Investigator 1	0.839	almost perfect	0.762	substantial	0.806	substantial	0.902	almost perfect
Investigator 2			0.769	substantial	0.668	substantial	0.934	almost perfect
Investigator 3					0.477	moderate	0.757	substantial
Investigator 4							0.879	almost perfect

0–0.2 slight agreement

0.21–0.4 fair

0.41–0.6 moderate

0.61–0.8 substantial

0.81–100 almost perfect

**Table 3:** Intraobserver reliability ICC of both methods in terms of angular and translational errors

*Angular error*					
	ICC		95% confidence interval		
Conventional	0.586	moderate	0.056	–	0.907
NAS	0.897	almost perfect	0.698	–	0.980
*Translational error*					
	ICC		95% confidence interval		
Conventional	0.619	substantial	0.030	–	0.920
NAS	0.938	almost perfect	0.816	–	0.988

**Fig. 6:**
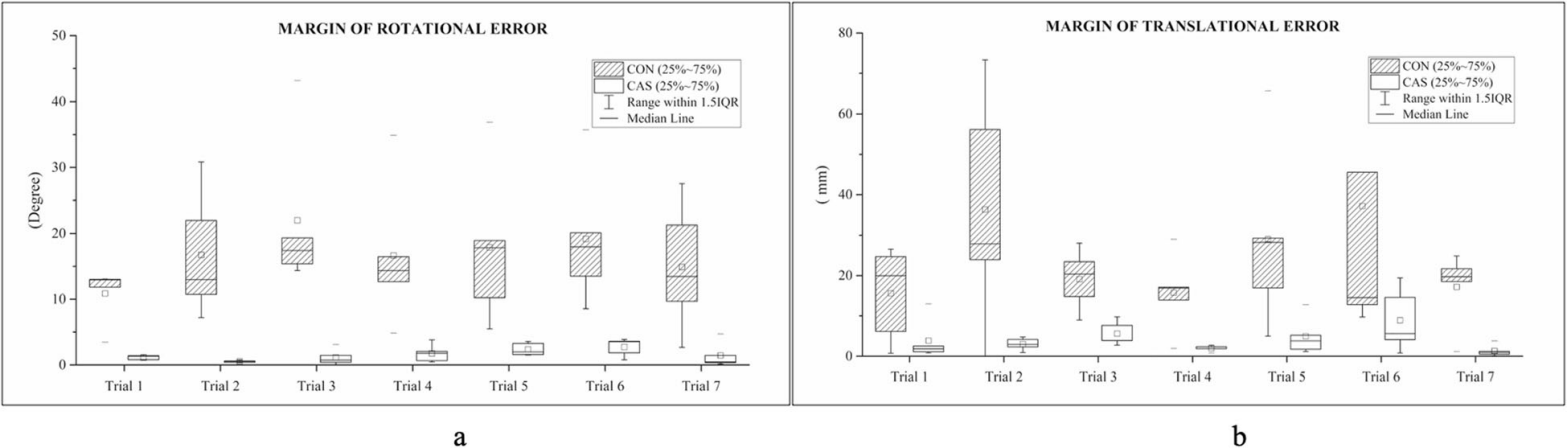
Differences in (**a**) angular and (**b**) translational errors by using navigation-assisted anchor fixation (CAS) and conventional method (CON)

Assessment of mean errors in each trial showed that the navigation-assisted method resulted in 91 and 83% fewer angular and translational errors, respectively (Fig. 6). Moreover, providing surgeons more information regarding the anchor position and its related anatomy could accelerate the learning curve, reducing the time required for acquiring skills on arthroscopic rotator cuff repair.

## Discussion

Most surgeons consider the orientation of suture anchor insertion for the repair of the subscapularis muscle as identical to that of the supraspinatus muscle. However, the footprints of both rotator cuff muscles are in different spatial orientations. The images provided by the arthroscope are intrinsically distorted, and the surgeons operate without any other visual feedback. Because surgeons are solely dependent on external anatomical appearance and arthroscope images, they must attempt optimal suture anchor insertion with limited information. Our findings suggest that the navigation system may help provide multiplanar visualization of the footprint, thereby helping surgeons perform these surgeries.

**Fig. 7:**
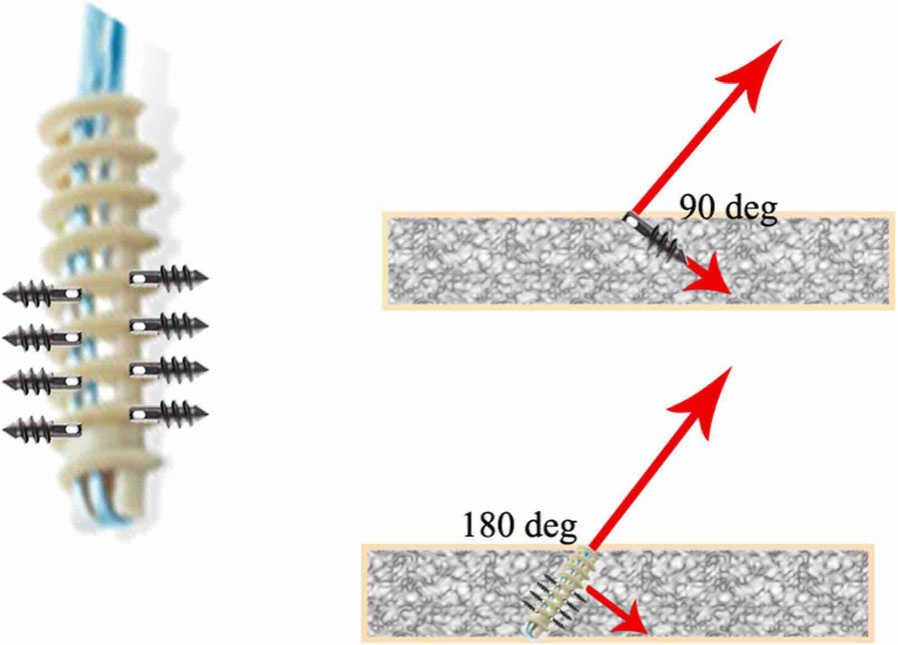
Cross-sectional views of anchor threads, showing that they act as small anchors that change the direction of the pull-out vector to 90°

Under ideal conditions, the 45° angle of anchor fixation can be determined from the thickness of the tendon and distance from the suture site to the anchor. Theoretically, this can result in better pull-out strength and less stress on the suture. Recent improvements to the anchor, including a change in thread, can change the axis of fixation by 90° (Fig. 7). Therefore, the pull-out strength must be recalculated to determine the ideal anchor angle for rotator cuff repair. Placing the angle of the anchor parallel or almost parallel to the suture can maximize the pull-out strength of the anchor [8]. We hypothesized that the angle and exact location of the anchors could be determined using navigation-assisted arthroscopic anchor fixation.

**Fig. 8:**
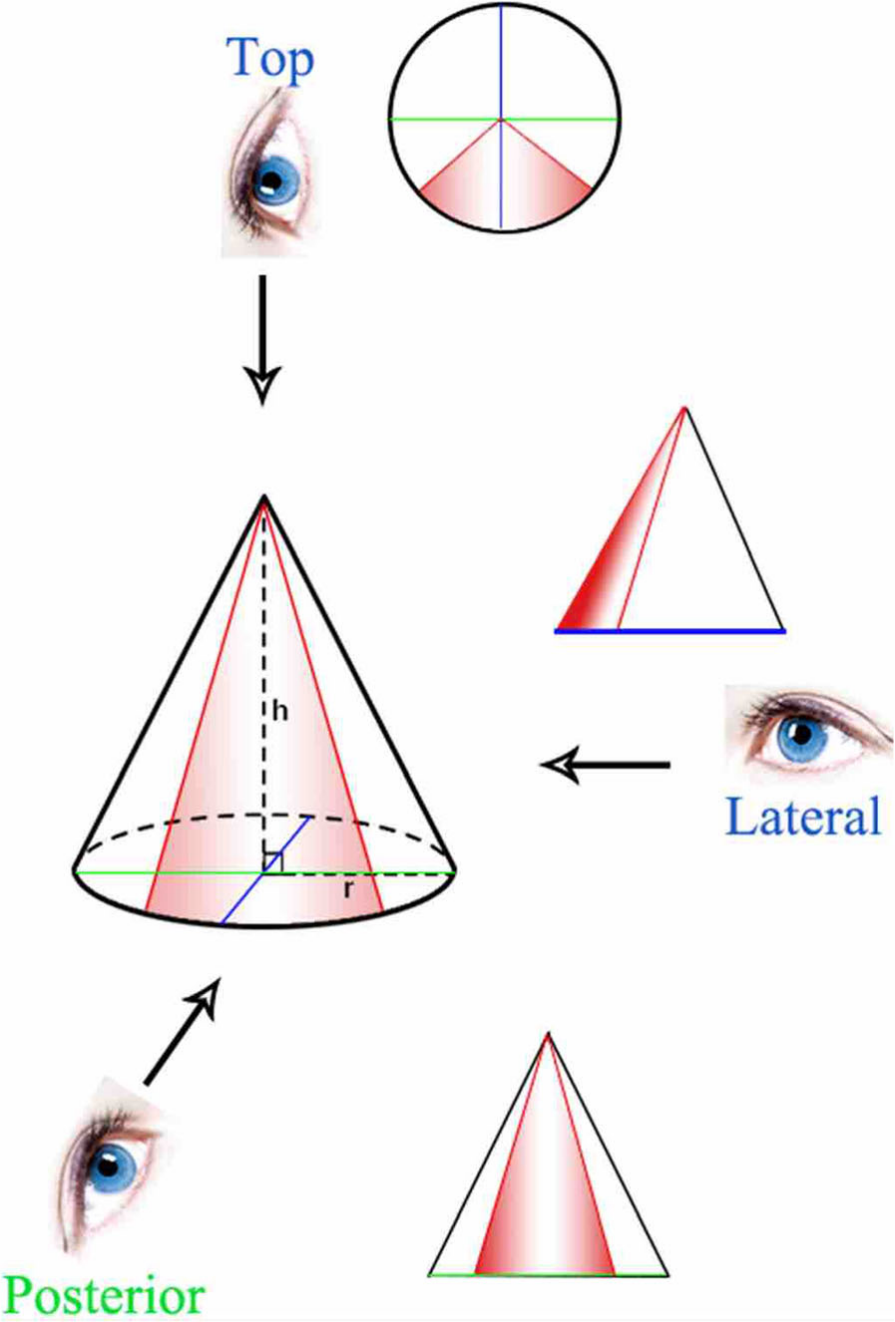
Cone phenomenon for arthroscopic instrument movement. The top of the cone represents the instrument portal. The red line represents the range of instrument or anchor, and the eyes show the viewing portals from the lateral, posterior, and top positions of the cone. Looking from the posterior or lateral portal can result in angles and locations of the anchor or instrument that can vary and be difficult to measure. The view from the top of the cone can better select the anchor or instrument location, and the angle can be determined based on the ratio of cone height to the anchor tip distance from the radial center, tan *θ* (*θ*= instrument angle to the cortical surface)

In basic operating settings for subscapularis repair, the use of the navigation system improved the ability of surgeons to orient the angle of suture anchor placement. Real-time feedback from the navigation system with multiplanar situational awareness of the tool on the humerus rendered the overall procedure arthroscopically more accurate. The interobserver reliability of ICCs showed reduced angular errors, indicating that angular orientation is more difficult to achieve than location. Although viewing through the posterior portal allows the anchor angle to be easily measured in the coronal plane, sagittal movement of the anchor is difficult to discern because of the use of a single arthroscope, which cannot measure depth. Rotating the instrument in the intraarticular space can better determine the angle when the instrument is moved along a plane perpendicular to the arthroscope. In contrast, determining the angle of the instrument is difficult when the instrument is moved along a plane parallel to the arthroscope. The angle and location of the anchor and instrument can vary and be difficult to measure when viewed from the posterior or lateral portal. In contrast, viewing from the top of the cone allows the location to be easily selected, with the angle determined from the cone height and distance from the anchor tip to the center of the radius (Fig. 8). Thus, placing the anchor insertion portal close to the viewing portal (proxy-viewing portal) can determine the accurate orientation of the anchor. The navigation system can measure the angle from multiple reference points in real time. Based on the cone phenomenon, the proxy-viewing portal close to the anchor insertion hole can result in a more accurate anchor angle and location than the posterior viewing portal.

Arthroscopic subscapularis repair is a well-established technique [18, 19]. The surgeon is frequently dependent on the view provided by the arthroscope to orient the insertion angle. However, the view provided by the 30° arthroscope is inherently distorted, and the total area of the footprint visible through the posterior portal in the gleno-humeral view is limited. The limited information reduces the surgeon’s ability to accurately determine the optimal angle for insertion of the suture anchor to stabilize the repair.

The present study showed that the navigated approach enhanced reliable results for all glenoid positions. Although optimizing anchor placement should theoretically improve the biomechanical behavior of the repaired area, these clinical data were not generated from the present study. This study aimed to assess whether intraoperative multiplanar visualization could reduce the number of errors from optimal angles. Furthermore, by determining the anchor location and angle, this method can be useful for randomized control studies or follow-up examinations related to anchor location and angle. The inclusion of quantitative information regarding the accuracy and duration of the procedure can enable this method to be utilized to evaluate a surgeon’s skills in arthroscopic shoulder surgery or to determine training objectives [20, 21].

The use of the navigation system altered the understanding of the surgeon while inserting the anchor into the subscapularis and supraspinatus footprint site, by making the surgeon more aware of the instrument location, along with their angular trajectory and penetration location of the anchor. The navigation system also provides information regarding the surrounding cortical thickness if the surgeon follows the trajectory depicted in the navigation experiment. Determining the correct orientation of the desired angle for subscapularis repair is difficult with the conventional method. As the number of trials increase, achieving the target angle and location remain difficult. The targeted angle and location may vary widely even when a plastic model is used. The use of the navigation system can better achieve a direction close to the predesigned angle and location, particularly for determining the reference guide for orientation of the tool while making the pilot hole or inserting the anchor.

We are aware that the navigation system is developed to assist in practical surgical application involving real patients. In practical surgical application, there may be two possible ways to secure the reference marker of the tracker. One is to attaching the marker on the patient’s skin with an elastic strap, which has been widely used for brain applications in commercial systems. Another option is to have a small stab skin incision, which allows K-wire fixation to the bone. Later, reference markers can be attached to the end of the K-wire. A similar method has been widely used in robotic arm-assisted surgery in orthopedic field (MAKO, Stryker®).

This study is limited by our use of a plastic model in the operative setting. No subscapularis tendon is attached to the lesser tuberosity of the plastic models, such that the direction of the subscapularis tendon could not be determined. We used plastic models (Arthrex, USA), which are is used for arthroscopic training in a dry laboratory [10, 11] to minimize anatomic variations reported in the literature [22, 23]. The current study tested novices as the participating surgeon, therefore it limit the generalization of the result to the more experienced surgeons. Despite that limitation, we believe the result of the study may help to reduce the learning curve for trainees.

## Conclusion

Navigation may help surgeons become more aware of the surrounding anatomy, resulting in better decisions regarding anchor angle and location from preoperative guides. The navigation system may better determine the spatial orientation of the footprint for the subscapularis. Navigation assistance can result in more accurate and reliable anchor fixation.

## Data Availability

The datasets used and/or analyzed in the present study are available from the corresponding author upon reasonable request.

## Abbreviations

ICC: Intraclass correlation coefficient

## References

[1] Kuntz AF, Raphael I, Dougherty MP (2014). Arthroscopic subscapularis repair. J Am Acad Orthop Surg.

[2] Burkhart SS, Brady PC (2006). Arthroscopic subscapularis repair: surgical tips and pearls a to Z. Arthroscopy : the journal of arthroscopic & related surgery : official publication of the Arthroscopy Association of North America and the International Arthroscopy Association.

[3] Burkhart SS, Lo IK (2006). Arthroscopic rotator cuff repair. J Am Acad Orthop Surg.

[4] Strauss E, Frank D, Kubiak E (2009). The effect of the angle of suture anchor insertion on fixation failure at the tendon-suture interface after rotator cuff repair: deadman's angle revisited. Arthroscopy : the journal of arthroscopic & related surgery : official publication of the Arthroscopy Association of North America and the International Arthroscopy Association.

[5] Burkhart SS (1995). The deadman theory of suture anchors: observations along a South Texas fence line. Arthroscopy : the journal of arthroscopic & related surgery : official publication of the Arthroscopy Association of North America and the International Arthroscopy Association.

[6] Burkhart SS (2009). Suture anchor insertion angle and the deadman theory. Arthroscopy : the journal of arthroscopic & related surgery : official publication of the Arthroscopy Association of North America and the International Arthroscopy Association.

[7] Burkhart SS (2014). The deadman theory is alive and well. Arthroscopy : the journal of arthroscopic & related surgery : official publication of the Arthroscopy Association of North America and the International Arthroscopy Association.

[8] Clevenger TA, Beebe MJ, Strauss EJ (2014). The effect of insertion angle on the pullout strength of threaded suture anchors: a validation of the deadman theory. Arthroscopy : the journal of arthroscopic & related surgery : official publication of the Arthroscopy Association of North America and the International Arthroscopy Association.

[9] Green RN, Donaldson OW, Dafydd M (2014). Biomechanical study: determining the optimum insertion angle for screw-in suture anchors-is deadman's angle correct?. Arthroscopy : the journal of arthroscopic & related surgery : official publication of the Arthroscopy Association of North America and the International Arthroscopy Association.

[10] Kholinne Erica, Gandhi Maulik J., Adikrishna Arnold, Hong Hanpyo, Kim Haewon, Hong Jaesung, Jeon In-Ho (2018). The Dimensionless Squared Jerk: An Objective Parameter That Improves Assessment of Hand Motion Analysis during Simulated Shoulder Arthroscopy. BioMed Research International.

[11] Jung K, Kang DJ, Kekatpure AL (2016). A new wide-angle arthroscopic system: a comparative study with a conventional 30 degrees arthroscopic system. Knee Surg Sports Traumatol Arthrosc.

[12] Kwak Jae-Man, Kholinne Erica, Gandhi Maulik, Adikrishna Arnold, Hong Hanpyo, Sun Yucheng, Koh Kyoung-Hwan, Jeon In-Ho (2019). Improvement of arthroscopic surgical performance using a new wide-angle arthroscope in the surgical training. PLOS ONE.

[13] Eliasziw M, Young SL, Woodbury MG (1994). Statistical methodology for the concurrent assessment of interrater and intrarater reliability: using goniometric measurements as an example. Phys Ther.

[14] Johansson KM, Adolfsson LE (2005). Intraobserver and interobserver reliability for the strength test in the constant-Murley shoulder assessment. J Shoulder Elb Surg.

[15] Shrout PE, Fleiss JL (1979). Intraclass correlations: uses in assessing rater reliability. Psychol Bull.

[16] Landis JR, Koch GG (1977). The measurement of observer agreement for categorical data. Biometrics.

[17] Karl JW, Redler LH, Tang P (2016). Delayed proximal migration of the radius following radial head resection for Management of a Symptomatic Radial Neck Nonunion Managed with radial head replacement: a case report and review of the literature. Iowa Orthop J.

[18] Park YB, Park YE, Koh KH (2015). Subscapularis tendon repair using suture bridge technique. Arthrosc Tech.

[19] Richards DP, Burkhart SS, Lo IK (2003). Subscapularis tears: arthroscopic repair techniques. Orthop Clin North Am.

[20] Cannon WD, Nicandri GT, Reinig K (2014). Evaluation of skill level between trainees and community orthopaedic surgeons using a virtual reality arthroscopic knee simulator. J Bone Joint Surg Am.

[21] Kirby GS, Guyver P, Strickland L (2015). Assessing arthroscopic skills using wireless elbow-worn motion sensors. J Bone Joint Surg Am.

[22] Curtis AS, Burbank KM, Tierney JJ (2006). The insertional footprint of the rotator cuff: an anatomic study. Arthroscopy : the journal of arthroscopic & related surgery : official publication of the Arthroscopy Association of North America and the International Arthroscopy Association.

[23] Ide J, Tokiyoshi A, Hirose J (2008). An anatomic study of the subscapularis insertion to the humerus: the subscapularis footprint. Arthroscopy : the journal of arthroscopic & related surgery : official publication of the Arthroscopy Association of North America and the International Arthroscopy Association.

